# Linking leaf veins to growth and mortality rates: an example from a subtropical tree community

**DOI:** 10.1002/ece3.2311

**Published:** 2016-07-29

**Authors:** Yoshiko Iida, I‐Fang Sun, Charles A. Price, Chien‐Teh Chen, Zueng‐Sang Chen, Jyh‐Min Chiang, Chun‐Lin Huang, Nathan G. Swenson

**Affiliations:** ^1^ Kyushu Research CenterForestry and Forest Products Research InstituteKurokami 4‐11‐16Chuo‐kuKumamoto860‐0862Japan; ^2^ Department of Natural Resources and Environmental StudiesNational Dong Hwa UniversityHualien974Taiwan; ^3^ School of Plant BiologyThe University of Western AustraliaCrawleyWestern Australia6009Australia; ^4^ Department of AgronomyNational Chung Hsing UniversityTaichung402Taiwan; ^5^ Department of Agricultural ChemistryNational Taiwan UniversityTaipei10617Taiwan; ^6^ Department of Life ScienceTunghai UniversityTaichung407Taiwan; ^7^ Laboratory of Molecular PhylogeneticsDepartment of BiologyNational Museum of Natural ScienceTaichung407Taiwan; ^8^ Department of BiologyUniversity of MarylandCollege ParkMaryland21148

**Keywords:** Functional trait, leaf venation, maximum height, nutrient, plant development and life‐history traits, relative growth rate, species distribution, specific leaf area, wood density

## Abstract

A fundamental goal in ecology is to link variation in species function to performance, but functional trait–performance investigations have had mixed success. This indicates that less commonly measured functional traits may more clearly elucidate trait–performance relationships. Despite the potential importance of leaf vein traits, which are expected to be related to resource delivery rates and photosynthetic capacity, there are few studies, which examine associations between these traits and demographic performance in communities. Here, we examined the associations between species traits including leaf venation traits and demographic rates (Relative Growth Rate, RGR and mortality) as well as the spatial distributions of traits along soil environment for 54 co‐occurring species in a subtropical forest. Size‐related changes in demographic rates were estimated using a hierarchical Bayesian approach. Next, Kendall's rank correlations were quantified between traits and estimated demographic rates at a given size and between traits and species‐average soil environment. Species with denser venation, smaller areoles, less succulent, or thinner leaves showed higher RGR for a wide range of size classes. Species with leaves of denser veins, larger area, cheaper construction costs or thinner, or low‐density wood were associated with high mortality rates only in small size classes. Lastly, contrary to our expectations, acquisitive traits were not related to resource‐rich edaphic conditions. This study shows that leaf vein traits are weakly, but significantly related to tree demographic performance together with other species traits. Because leaf traits associated with an acquisitive strategy such as denser venation, less succulence, and thinner leaves showed higher growth rate, but similar leaf traits were not associated with mortality, different pathways may shape species growth and survival. This study suggests that we are still not measuring some of key traits related to resource‐use strategies, which dictate the demography and distributions of species.

## Introduction

The importance of linking morphology to performance has been recognized in ecology and evolution (Arnold 1983), but, the aspects of plant form and function that determine individual species performance, such as growth and mortality rates, within and across biomes remain poorly understood (e.g., Paine et al. 2015). This lack of understanding not only applies to highly diverse tropical and subtropical forests, but also to less diverse temperate forests (Martinez‐Vilalta et al. 2010). Trait–performance or community trait dispersion studies within or across diverse tree communities have used primarily easily measured plant functional traits, which are generally indirectly related to the physiological rates of interest (Poorter et al. 2008; Wright et al. 2010). This is especially true for leaf morphological traits that are weakly correlated with demographic rates across co‐occurring diverse species compared to other organ's traits such as wood density and plant maximum height (Poorter et al. 2008; Martinez‐Vilalta et al. 2010; Wright et al. 2010; Ruger et al. 2012; Lasky et al. 2013; Iida et al. 2014a).

Recent studies have demonstrated that easily measured leaf traits are poor predictors of interspecific variation in growth and survival rates within individual communities (Poorter et al. 2008; Martinez‐Vilalta et al. 2010; Wright et al. 2010; Ruger et al. 2012; Lasky et al. 2013; Iida et al. 2014a) and across forests worldwide (Paine et al. 2015). Based on the idea that interspecific differences in resource allocation strategies to stems, leaves, and seeds underlie function and performance, traits such as maximum height, wood density, specific leaf area (SLA), and seed mass have been proposed as important functional traits (e.g., Westoby et al. 2002). Communitywide studies have confirmed modest links between traits such as maximum height and wood density, with performance measures such as growth and survival (Poorter et al. 2008; Wright et al. 2010; Iida et al. 2014a,b). However, SLA, an index of the resource allocation strategy of a single leaf, and one of the most commonly measured traits, is typically very weakly correlated or not correlated with demographic rates. The failure of easily measured leaf functional traits to strongly predict demographic rates across co‐occurring tree species is due to a suite of issues such as size‐related changes in the trait–demography relationships (Iida et al. 2014a) and their indirect or loose relationships with photosynthetic capacity and water resource utilization strategies (Reich 2014). However, direct measurements of physiological traits are still challenging for diverse tree communities. Thus, trait‐based ecology needs to identify those traits that are simultaneously feasible to measure in diverse assemblages and are directly linked to physiological rates.

While the study of variability in leaf traits has long been a central research theme in ecology, the role of leaf veins has recently garnered more attention (Blonder et al. 2011; Price et al. 2011b; Sack and Scoffoni 2013; Li et al. 2015) and may provide a pathway forward for linking leaf traits with demographic rates. The flow of water from roots, through shoots and leaves to the surrounding air, follows a pathway of increasingly negative water potential known as the soil–plant–atmosphere continuum (Sperry et al. 2003). This hydraulic flow path bridges two key processes: the acquisition of water by roots and the loss of water as transpiration by leaves. Because of the shared pathway of transpirational water loss and photosynthetic carbon gain, through stomates, any investment in leaf structure that increases hydraulic transport, while also maintaining a balance between safety and efficiency (Sperry et al. 2008), will potentially benefit from greater carbon uptake. Most of the flow path is characterized by bulk flow in specialized conduits known as xylem with relatively high conductance to water. Upon reaching the terminal veins in leaves, water must diffuse through the low conductance mesophyll and keep the mesophyll hydrated to allow stomates to remain open (Buckley 2005). Compared to bulk flow, diffusion is very slow, and thus, the spacing between veins has a strong influence on the rate at which water can be supplied to the mesophyll tissue. All else being equal, a close spacing of veins can support a faster delivery of water and ultimately support faster photosynthetic and growth rates (Brodribb et al. 2007).

Across species, leaf vein networks display tremendous variety in their form (Price et al. 2011b, 2013). Leaf vein networks and vein length per unit area (VLA), also referred to as leaf vein density, in particular, have been linked to whole leaf conductance (Cochard et al. 2004; Sack and Holbrook 2006), photosynthetic rates (Brodribb et al. 2007), species diversification rates (Brodribb and Feild 2010; Brodribb et al. 2010) and have been suggested as a proxy for paleoclimatic variability (Uhl and Mosbrugger 1999). Of particular relevance to our work that focuses on growth and mortality, variability in VLA has been linked to differences in leaf physiology at early ontogenetic stages (Sack and Frole 2006). Specifically, it is frequently postulated that tree species demography can be understood by determining where a species falls along a continuum between conservative and acquisitive resource‐use strategies determined by their functional traits (e.g., Grime 2001; Sterck et al. 2011). In the case of tree communities, light use strategies are expected to be particularly important. However, identifying and quantifying leaf traits that are indicative of these strategies and demonstrating their relationship with demographic rates has been challenging. Leaf vein networks and VLA, in particular, show promise as integrative traits with considerable potential to inform us regarding differences in plant physiology and light use strategies (Sack and Scoffoni 2013; Li et al. 2015). Despite this promise, VLA and other venation traits have yet to be widely incorporated into plant community ecology or explicitly linked to individual performance (i.e., growth) at the community scale.

The potential importance of leaf venation with respect to demographic rates should also affect community assembly and species coexistence, but robust tests of associations of vein traits with growth and mortality across co‐occurring species are uncommon. Further, relatively little is known regarding how vein traits that are directly related to photosynthetic capacity are spatially distributed along important local‐scale abiotic gradients such as soil nutrient gradients that play a strong role in dictating community composition through plant–environment interactions mediated by traits. For example, traits related to high photosynthetic capacities are expected to be associated with soils with higher nitrogen content. Specifically, species with acquisitive leaf venation strategies that increase water supply to the site of photosynthesis may also require more nitrogen, a key component of Rubisco, to maintain higher photosynthetic capacities. Thus, it is likely that leaf venation and soil nitrogen content will be related to one another and consequently shape species distributions. Ultimately, tests are needed that (1) explicitly quantify the relationship between leaf vein traits and growth and mortality rates; (2) characterize how these relationships fare compared to those for commonly measured traits of leaf and wood, and (3) examine how leaf vein traits are distributed along local‐scale abiotic gradients.

To address these questions, we examined the interspecific relationships between species traits, including leaf vein traits and demographic rates across 54 species in a Taiwanese subtropical rainforest across a wide range of size classes. We also quantified the association between these traits and soil environmental variables. Specifically, we quantified four leaf venation network traits together with eight other commonly measured functional traits to determine whether vein traits have stronger linkage. We estimated relative growth rate and mortality rate as a function of stem diameter to consider intraspecific size‐dependent changes in growth and mortality (e.g., Iida et al. 2014a,b). Because size‐dependent changes in the relationship between traits and demographic rates are reported to be largely due to size‐dependent changes in demographic rates and trait–demography relationships are clear for juvenile tree leaves in this site (Iida et al. 2014a), we consider the size‐dependent change in demographic rates and applied leaf traits obtained from juvenile trees in this study. We hypothesized that leaf vein traits may underlie conservative and acquisitive species resource‐use strategies through their effects on hydraulic supply and photosynthesis, which in turn influence demographic rates. Acquisitive species tend to show high‐resource acquisition and high growth rates and are successful in high‐resource habitats; in contrast, conservative species tend to show high‐resource conservation, high stress tolerance, and low mortality, especially in low‐resource habitats (Grime 2001; Russo et al. 2008). Therefore, we predicted that (1) leaf vein traits are closely related to rates of growth and mortality. Specifically, higher VLA or thicker veins supporting more efficient hydraulic transport allow higher gas exchange rates and therefore higher growth and mortality rates; and (2) distributions of leaf vein traits are also related to soil nutrient variables by reflecting their performance based on their physiological function. Specifically, the distribution of acquisitive vein traits should be correlated with resource‐rich habitats such as those containing high soil nitrogen and phosphorus levels.

## Materials and Methods

### Study site and species

We conducted this study in a tree community in the 25‐ha Fushan Forest Dynamics Plot (FDP) in the subtropical rainforests of northern Taiwan (24°45′40″N, 121°33′28″E, 600–733 asl) that receives a mean annual rainfall of 4271 mm (Su et al. 2007). The Fushan FDP was established in 2003–2004, and all trees which have a stem diameter, *D*, equal to or greater than 1 cm at breast height (1.3 m) were tagged, measured, identified, and mapped within the 500 m by 500 m plot area following the Center for Tropical Forest Science census protocol (Condit 1998). The second census was conducted in 2008–2009, and 107 woody species were recorded. For this study, we used 54 tree species with a wide range of maximum tree height ranging from 2.6 to 28.6 m.

### Trait measurements

We collected 1–3 intact and exposed leaves or leaflets from the outer crown for individual trees (1 cm ≤ *D *<* *3 cm) for 54 species (average 8 trees ranging from 1 to 23 trees per species) (Table S1). We measured leaf area (LA; cm^2^), specific leaf area (SLA; cm^2^ g^−1^), leaf thickness (mm), and succulence (gH_2_O cm^−2^) according to Cornelissen et al. (2003). For 48 of the 54 species, total organic nitrogen mass per unit leaf mass (Nmass, %) and total organic phosphorus mass per unit leaf mass (Pmass, %) were determined by two microplate methods (Huang et al. 2011; See detail in Iida et al. 2014a). The species mean wood density (WD; g cm^−3^) was calculated by the dry weight divided by wood volume using wood segments obtained from randomly selected five individuals of each species for 43 species outside the 25 ha FDP. Maximum height (Hmax; m) was estimated as an average value using the heights of the six largest trees of each species within the 25‐ha plot. A full description of the trait measurements is given in Appendix S1.

The leaf clearing for quantification of venation networks followed a protocol adapted from Gardner (1975) which uses a 5% NaOH solution as the principal clearing agent. We selected one intact and exposed leaf per species obtained from the outer crown. Species‐level differences should be easily and robustly detected even at low sample sizes given the large differences in venation networks among species. We tested this using VLA data for the genus *Banksia* (Proteaceae) collected by co‐author CAP and a taxonomically nested variance partitioning analysis. This analysis showed that 70.08% of the variance in VLA for 100 species in this single genus *Banksia* (Proteaceae) was explained by interspecific differences and 29.92% of the variation was within species. Once cleared, leaf sections were stained with a 1% safranin solution. Most leaf sections were stained for 1–3 min and then placed in a 95% ethanol solution until the veins were sufficiently destained to provide good contrast with surrounding lamina tissue. Following staining, leaf subsections were photographed away from the major veins, using a Nikon microscope (model: SMZ800) at 20× magnification.

The LEAF GUI software was used to obtain relevant network and areole (an area between veins) geometric and topological information from all photographed leaf vein images (Price et al. 2011a,b). After each stained leaf image was transformed to a binary image (white veins on black background), vein network topological and geometric information was extracted from the binary image of each leaf subsection. A full description of LEAF GUI can be found in Price et al. (2011a,b).

We utilized four different measures of vein geometry, vein length per area (VLA), the distance to the nearest areole (DA), the distance to the nearest vein (DV), and the vein areole distance ratio (VADR). The first, VLA is well established in the literature and has been shown to be correlated with a number of leaf morphological and physiological traits (Sack and Scoffoni 2013). DA is a proxy for vein width and is the average of the distance from each vein pixel to the nearest nonvein pixel. It is likely that the flow of water through vein increases with vein width, thus measures such as DA that begin to capture this additional source of variability may prove useful. DV is similarly calculated as the mean of the distances from each areole pixel to the nearest vein pixel. DV will be correlated with areole size and the distance over which fluid must diffuse upon leaving the vessels. While VLA has proven a useful measure of influence of vein geometry on physiology, it may not capture variability in vein and/or areole sizes well. For example, one could imagine two leaves with identical VLA but very different vein or areole thicknesses. As VADR represents the ratio of the DV to DA, it has the potential to capture both conductance and diffusion effects in a more robust way than VLA alone where high VADR values should be associated with lower photosynthetic capacity compared to low VADR values. Thus, VADR is potentially a more integrative measure of the complex structure of vein networks.

### Soil variables within the plot

We estimated four soil variables, pH in water (pH_water), total soil organic carbon (OC), available nitrogen (AV_N), and available phosphorus (AV_P) based on soil samples collected from 80 quadrats, which were distributed randomly over the whole 25 ha FDP. Kriging in the geostatistic software Surfer 7.0 was used to produce the soil distribution map for the plot on the scale of 20 × 20 m. A full description of the soil measurements is given in Appendix S1.

### Growth and mortality rates

To estimate size‐related changes in growth and mortality rates, we estimated relative growth rate (RGR) and mortality rate as a function of stem diameter by applying a hierarchical Bayesian model as originally described in Iida et al. (2014b).

To describe the nature of the declines in RGR with tree size (Ryan et al. 1997; Mencuccini et al. 2005; Rose et al. 2009), we assumed that the RGR of *i*th individual tree, *R*_*i*_, follows a linear function of the natural logarithm of the stem diameter at the first census *D*_1*i*_ of individual tree *i* with parameters for species *j* (*r*_*kj*_ *, k *=* *1, 2) as *R*_*i*_ = *r*_1*j*_ + *r*_2*j*_ ln(*D*_1*i*_). The logarithm of the stem diameter at the second census *D*_2*i*_ was assumed to be the sum of the logarithm of stem diameter at the first census *D*_1*i*_ and the product of *R*_*i*_ and the census interval of tree *i*,* t*_2,*i*_ − *t*_1,*i*_, as ln(*D*_2*i*_) = ln(*D*_1*i*_) + *R*_*i*_(*t*_2*i*_ − *t*_1*i*_).

To describe nonlinear size‐dependent changes in mortality such as U‐shaped curves with increasing size (e.g., King et al. 2006), we developed a hierarchical Bayesian model of mortality based on Ruger et al. (2011) and Iida et al. (2014b). The observation of whether a tree individual *i* survived through the census period (*S*_*i*_ = 1) or not (*S*_*i*_ = 0), *S*_*i*_ is assumed to follow a Bernoulli distribution with the predicted probability of survival *p*_*i*_ as *S*_*i*_ ~ Bernoulli (*p*_*i*_). The probability of survival of the *i*th individual tree (*p*_*i*_) was estimated from an instantaneous mortality rate of an individual tree *i* (*M*_*i*_, year^−1^) and adjusted for the time period between the first census (*t*_1*i*_) and the second census (*t*_2*i*_) as *p*_*i*_ = exp[−*M*_*i*_(*t*_2*i*_ − *t*_1*i*_)]. *M*_*i*_ was predicted as a function of the stem diameter of individual *i* at the first census, *D*_1*i*_ as ln(*M*_*i*_) = *m*_1*j*_ + *m*_2*j*_ln(*D*_1*i*_) + *m*_3*j*_ *D*_1*i*_.

We applied the RGR and mortality models to 61,273 and 75,071 individual trees census data between 2003 and 2008 belonging to the 54 species, respectively. We estimated the probability distributions of the parameters in each model. Full descriptions of the models are given in Appendix S2. Sampling from the probability distribution of all parameters in models for RGR and mortality was performed using the Markov chain Monte Carlo method in WinBUGS 1.4.3 (Spiegelhalter et al. 2003).

### Relationships between traits, demographic rates, and soil variables

To examine the relationships between species traits and between individual traits and demographic rates, we calculated Kendall's correlation coefficient, *τ*. To determine whether phylogenetically independent contrasts were necessary for our correlation analyses (Felsenstein 1985), we quantified the phylogenetic signal in the four leaf venation traits (VLA, DA, DV, and VADR) and the other 8 functional traits (LA, SLA, succulence, thickness, Nmass, Pmass, WD, and Hmax) in this study. The phylogeny used for these analyses was generated by pruning the 54 study species out of a large molecular phylogeny inferred using the DNA barcode regions *RbcL*,* matK,* and *trnH‐psbA* (Erickson et al. 2014). The sequence data for these species came from the Fushan forest dynamics plot. Phylogenetic signal was measured using Blomberg's K statistic (Blomberg et al. 2003), and *P*‐value was estimated using a Monte Carlo test using the R‐package “*picante*.” Because the traits, including leaf vein traits, did not have significant phylogenetic signal, we did not utilize phylogenetically independent contrasts in our correlation analyses below.

The trait–demographic rate correlations utilized the whole probability distributions of demographic rates (RGR and mortality) at a given stem diameter (reference *D*) at 1‐cm interval from 1‐cm stem diameter. The probability distribution of Kendall's correlation coefficient *τ* was estimated from a correlation test between the probability distribution of demographic rates at a reference *D* and each species trait. We determined that the Kendall's correlation was significant if the 95% interval of the probability distribution of correlation coefficient does not include the zero. For species traits, which are significantly correlated with Hmax/WD (Table 1), Kendall's partial rank correlations were used to remove the effect of Hmax/WD on demographic rates because Hmax correlates with relative growth rate and WD correlates with mortality (Iida et al. 2014a). We analyzed correlations for species whose 95th percentile maximum stem diameter (D95) is larger than reference *D*. D95 was estimated for trees of the subpopulation whose *D* was equal to or >10% of the observed maximum D of a population to reduce the effect of population size (King et al. 2006). Because of selection by D95, the number of species used in correlation analysis declined with increasing reference *D* due to the dropping out of short‐statured species from the comparison. Species were included in the correlation analysis when D95 was larger than each reference *D*. Additionally, to confirm whether size‐related change in correlations was not only due to size‐related changes in sample size (i.e., the number of species compared), we applied the same analyses for top 20 large‐statured species.

**Table 1:** Kendall's correlation coefficients between pairs of 12 species traits

	VLA (*τ*)	DA (*τ*)	DV (*τ*)	VADR (*τ*)	LA (*τ*)	SLA (*τ*)	Succulence (*τ*)	Thickness (*τ*)	Nmass (*τ*)	Pmass (*τ*)	WD (*τ*)	Hmax (*τ*)
VLA		0.34***	−0.87***	−0.63***	−0.03	−0.16	−0.36***	−0.18	0.02	0.01	0.36***	0.27**
DA	0.34***		−0.45***	−0.7***	−0.05	−0.34***	−0.17	0.04	−0.23*	−0.16	0.19	0.07
DV	−0.87***	−0.45***		0.74***	0.05	0.17	0.36***	0.17	0.02	−0.01	−0.33**	−0.24*
VADR	−0.63***	−0.7***	0.74***		0.04	0.27**	0.27**	0.04	0.15	0.08	−0.3**	−0.2*
LA	−0.03	−0.05	0.05	0.04		0.12	0.23*	0.05	0.09	0.07	−0.34**	0.03
SLA	−0.16	−0.34***	0.17	0.27**	0.12		−0.08	−0.49***	0.49***	0.3**	−0.29**	−0.12
Succulence	−0.36***	−0.17	0.36***	0.27**	0.23*	−0.08		0.54***	−0.17	0.01	−0.21	−0.16
Thickness	−0.18	0.04	0.17	0.04	0.05	−0.49***	0.54***		−0.38***	−0.17	0.05	−0.05
Nmass	0.02	−0.23*	0.02	0.15	0.09	0.49***	−0.17	−0.38***		0.42***	−0.1	−0.06
Pmass	0.01	−0.16	−0.01	0.08	0.07	0.3**	0.01	−0.17	0.42***		−0.19	−0.09
WD	0.36***	0.19	−0.33**	−0.3**	−0.34**	−0.29**	−0.21	0.05	−0.1	−0.19		0.22*
Hmax	0.27**	0.07	−0.24*	−0.2*	0.03	−0.12	−0.16	−0.05	−0.06	−0.09	0.22*	

Correlations coefficients by Kendall's correlation for each pair of two traits were shown. Twelve species traits include four leaf venation traits of vein density (VLA), the mean distance to nearest areole (DA), the mean distance to nearest vein (DV), and the vein areole distance ratio (VADR), and six leaf traits of juvenile trees, leaf area (LA), specific leaf area (SLA), succulence, thickness, and mass‐based nitrogen and phosphorus contents (Nmass and Pmass), and wood density (WD), maximum height (Hmax).

Asterisk shows level of significant: ***P < 0.001, **P < 0.01, *P < 0.05.

To analyze the association between traits and soil environmental variables, we calculated the mean soil environmental variables for all of the individuals for each species and correlated these values with the trait data. All statistical analyses were conducted using the statistical computing program R 2.14.2 (R Development Core Team 2015).

## Results

### Trait–trait relationships

Species mean values for the twelve traits, including four vein traits (vein length per area [VLA], the mean distance to nearest areole [DA], the mean distance to nearest vein [DV], and the vein areole distance ratio [VADR]) were more labile than expected from a Brownian Motion model of trait evolution (*K* < 0.01; *P*‐value > 0.05; Table S2), which indicates a lack of phylogenetic signal. Therefore, we did not utilize phylogenetically independent contrasts in the analyses below. The four leaf vein traits were significantly correlated with each other (Table 1). The VLA was positively correlated with wood density (WD) and maximum height (Hmax) and negatively correlated with leaf succulence. The distance to nearest areole, DA, a measure of average vein thickness, was negatively correlated with specific leaf area (SLA) and mass‐based nitrogen content of leaf (Nmass). The distance to nearest vein, DV, a measure of areole size, was positively correlated with succulence and negatively correlated with WD and Hmax. The vein areole distance ratio, VADR, which is an index related to the water supply per areole area, was positively correlated with SLA and succulence and negatively correlated with WD and Hmax.

### Relationships between traits and demographic rates

**Figure 1:**
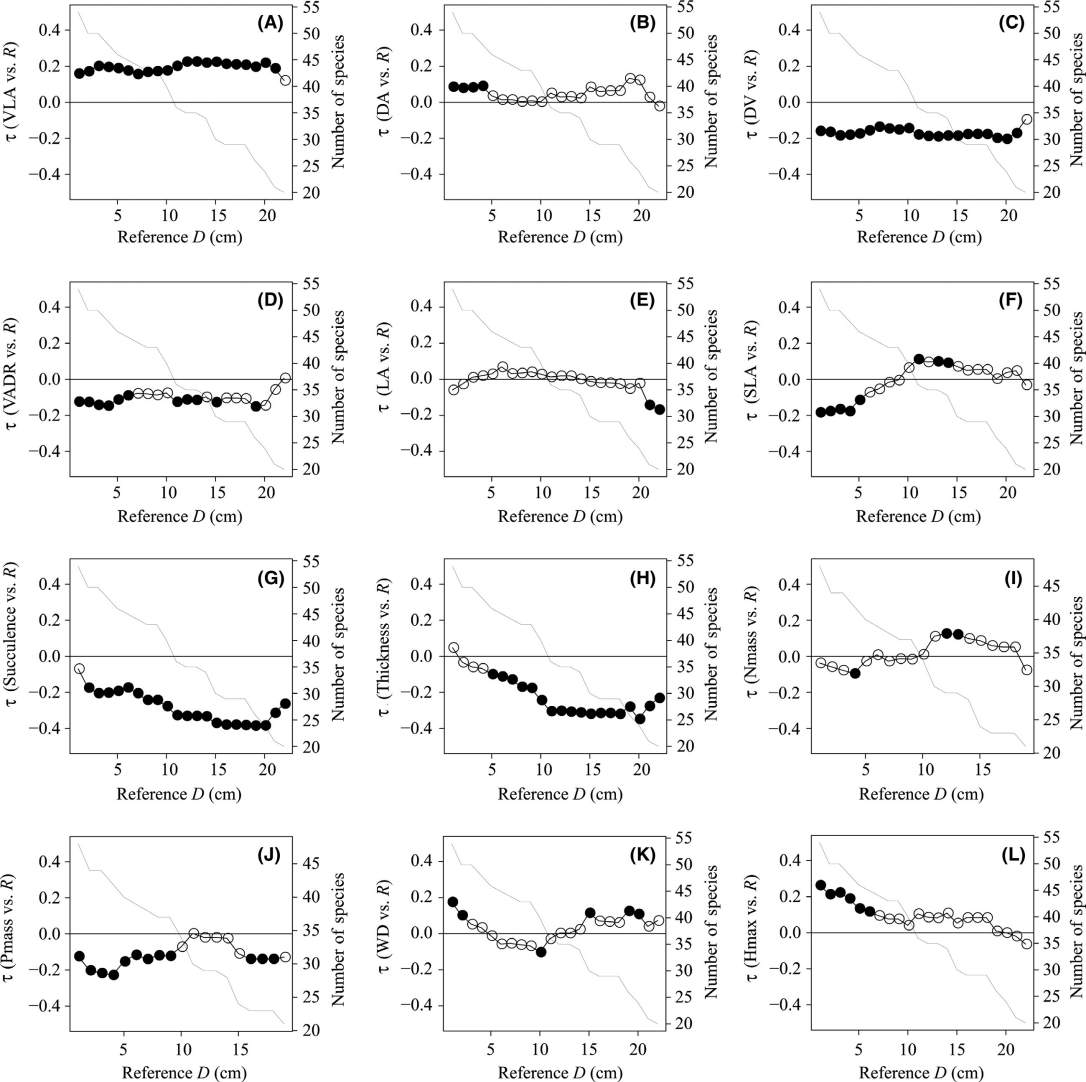
Correlation coefficients between 12 species traits and relative growth rates, *R* at 1‐cm stem diameter. Species traits includes four leaf vein traits of (A) vein density (VLA), (B) the mean distance to nearest areole (DA), (C) the mean distance to nearest vein (DV), and (D) the vein areole distance ratio (VADR), and other six leaf traits of (E) leaf area (LA), (F) specific leaf area (SLA), (G) succulence, (H) thickness, and (I) mass‐based nitrogen and (J) phosphorus (Nmass and Pmass), and (K) wood density (WD) and (L) maximum height (Hmax). Each value of correlation coefficient, tau indicates the median of probability distribution of tau. In the case, that 95% interval of probability distribution does not include the zero, the correlation between species trait and relative growth rate is significant and then circle was filled. The downward line shows the decline of number of species compared. Species whose D95 is less than reference D was excluded from comparison and correlations were applied until the reference D which included 20 species for comparison.

Size‐related changes in the correlations between species traits and RGR varied largely (Fig. 1). Of the four leaf vein traits, vein density (VLA) and the mean distance to the nearest vein (DV) were constantly significantly correlated with RGR for a wide range of reference stem diameter. VLA was positively and DV, a proxy of areole size, was negatively correlated with RGR from 1‐cm up to 21‐cm stem diameter (Fig 1A and C). The distance to the nearest areole (DA), a proxy for vein width, was significantly positively correlated with RGR only when trees are small at 1–4 cm diameter (Fig. 1B). The vein areole distance ratio (VADR), a measure of the complex structure of vein networks, was negatively correlated with RGR at small sizes < 7 cm and changed to nonsignificant at some sizes more than 7 cm (Fig. 1D). These results suggest that species with dense and/or wider veins, or smaller areoles tend to have faster RGR.

Of the six other leaf traits, leaf succulence and thickness were negatively correlated with RGR for a wide range of sizes (Fig. 1G and H) and Pmass was negatively correlated with RGR up to 9‐cm stem diameter, suggesting that species with more succulent, phosphorus‐rich or thicker leaves tend to have faster RGR. Species maximum height was positively correlated with RGR only at small size classes less than 7‐cm stem diameter (Fig. 1L), suggesting that more large‐statured species tend to have faster RGR at small sizes.

**Figure 2:**
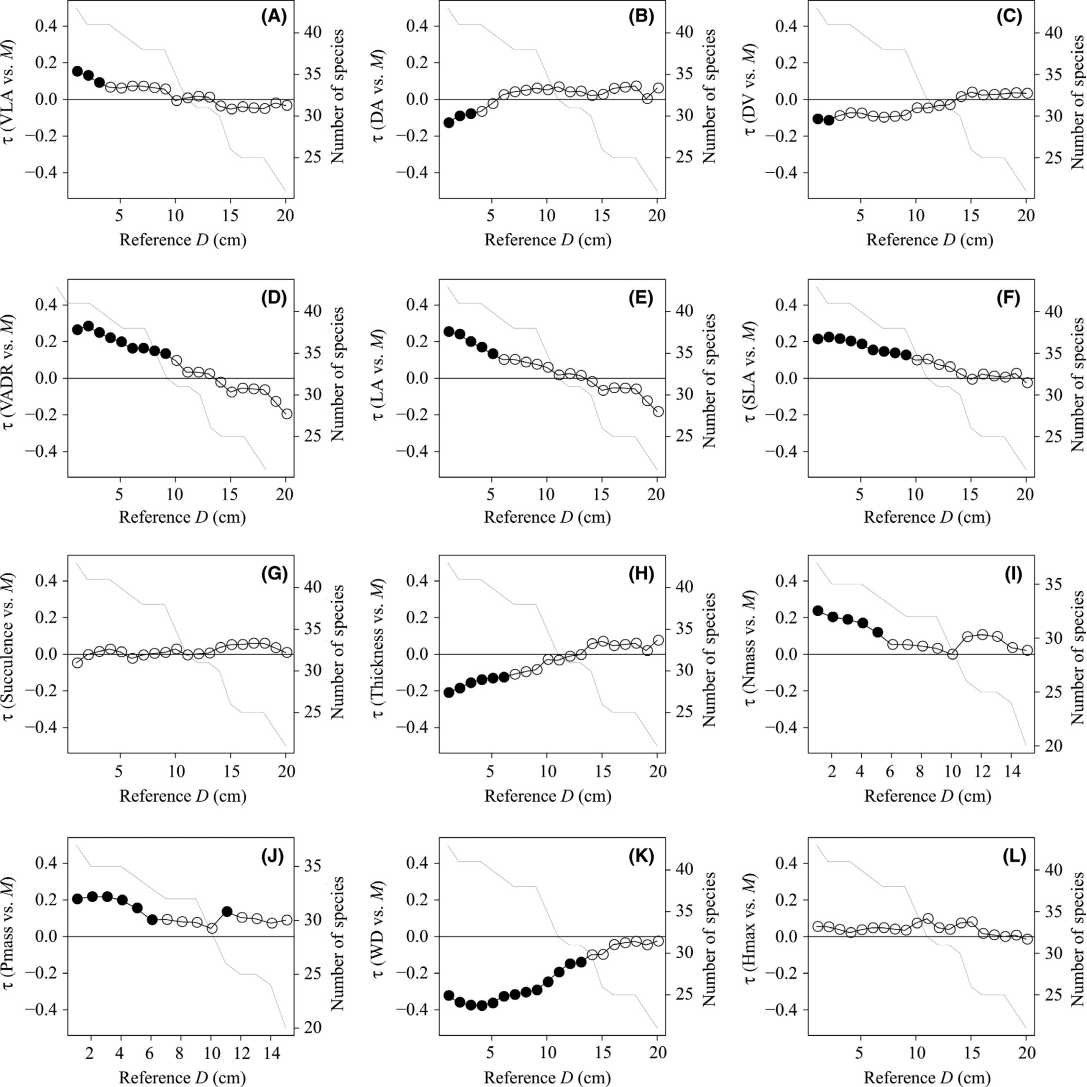
Correlation coefficients between 12 species traits and mortality rate, *M* at 1‐cm stem diameter. Species traits includes four leaf vein traits of (A) vein density (VLA), (B) the mean distance to nearest areole (DA), (C) the mean distance to nearest vein (DV), and (D) the vein areole distance ratio (VADR), and other six leaf traits of (E) leaf area (LA), (F) specific leaf area (SLA), (G) succulence, (H) thickness, and (I) mass‐based nitrogen and (J) phosphorus (Nmass and Pmass), and (K) wood density (WD) and (L) maximum height (Hmax). Each value of correlation coefficient, tau indicates the median of probability distribution of tau. In the case, that 95% interval of probability distribution does not include the zero, the correlation between species trait and mortality rate is significant and then circle was filled. The downward line shows the decline of number of species compared. Species whose D95 is less than reference D was excluded from comparison and correlations were applied until the reference D which included 20 species for comparison.

Significant correlations between species traits and mortality rates were found only at small size classes (Fig. 2). VLA and VADR were positively and DA and DV were negatively correlated with mortality rates, suggesting that species with denser or narrower vein, or smaller window tend to show higher mortality rates at 1‐ to 3‐cm stem diameters and species with lower photosynthetic capacity tend to show higher mortality rate up to 9‐cm stem diameter. Leaf area (LA), specific leaf area (SLA), and mass‐based nitrogen and phosphorus contents (Nmass and Pmass) were positively and leaf thickness was negatively correlated with mortality rates at small size classes, suggesting that species with larger, cheaper, thinner, nitrogen‐rich, or phosphorus‐rich leaves tend to show higher mortality at small sizes. Wood density was negatively correlated with mortality rates up to 13‐cm stem diameter but Hmax was not significantly correlated with mortality rates at any sizes.

Correlations between species traits and RGR/mortality changed with increasing size for the 20 largest statured species (15 largest statured species for leaf chemical traits) for which the species number did not change with increasing a given stem diameter (Figs S1 and S2). These results indicate that size‐related change in correlations was not only due to decrease in species number but also due to the size‐related change in demographic rates.

### Relationships between traits and soil variables

We quantified the correlation between the species traits and their average soil environment. We found that VLA was negatively and DV was positively weakly significantly correlated with species‐average available nitrogen (AV_N) (Table 2). DA and VADR were not significantly correlated with species‐specific soil environment. Nmass was positively correlated with available phosphorus (AV_P), and Pmass was negatively correlated with available nitrogen (AV_N). WD was positively correlated with total soil organic carbon (OC).

**Table 2:** Kendall's correlation coefficients between 12 species traits and four soil variables

	pH_water (*τ*)	OC (*τ*)	AV_N (*τ*)	AV_P (*τ*)
VLA	−0.05	−0.08	−0.22*	−0.05
DA	−0.05	0.05	0	0.05
DV	0.02	0.08	0.21*	0.01
VADR	0.03	0.01	0.13	−0.05
LA	−0.02	−0.14	−0.09	0.06
SLA	0.1	−0.1	−0.11	0.08
Succulence	0.12	−0.06	0.1	−0.04
Thickness	0.02	0.04	0.13	−0.06
Nmass	0.02	−0.07	−0.15	0.27**
Pmass	0.11	−0.16	−0.24*	0.07
WD	−0.05	0.21*	0.11	0.17
Hmax	0.1	−0.08	0.02	−0.06

Twelve species traits include four leaf venation traits of vein density (VLA), the mean distance to nearest areole (DA), the mean distance to nearest vein (DV), and the vein areole distance ratio (VADR), and wood density (WD), maximum height (Hmax), and six leaf traits of juvenile trees, leaf area (LA), specific leaf area (SLA), succulence, thickness, and mass‐based nitrogen and phosphorus contents (Nmass and Pmass). Four soil variables include pH in water (pH_water), total soil organic carbon (OC), available nitrogen (AV_N), and available phosphorus (AV_P).

Asterisks show the level of significant: **P < 0.01, *P < 0.05.

## Discussion

Linking organismal traits to demographic rates is critical for our ability to understand and predict the structure and dynamics of ecological communities upon the basis of trait data. Where tree species fall along a continuum from conservative strategies best fit for low‐resource conditions to acquisitive strategies best fit for high‐resource conditions (Grime 2001; Russo et al. 2008) can be explained in terms of association between functional traits and demographic rates of growth and morality. Recent work has demonstrated that there may be a strong relationship between leaf venation networks, photosynthetic capacity, and shade tolerance (e.g., Sack and Frole 2006). Here, we have performed the first communitywide analysis of whether there is a significant relationship between leaf venation networks and tree demographic rates, and the first test of the spatial variation in leaf venation network composition in a community in a Taiwanese subtropical forest dynamics plot. The results show that leaf venation traits are significant predictors of growth rate as well as other species traits reported but leaf venation traits did not show relationships with species‐average edaphic condition to support acquisitive and conservative resource‐use strategies.

### Leaf venation and demographic rates

All twelve traits, including the four leaf vein traits, were more labile than expected from a Brownian Motion model of trait evolution. In other words, they lacked phylogenetic signal. The vein length per area result contrasts with a previous analysis using 96 species from a broader taxonomic sampling that showed phylogenetic signal (Walls 2011). A likely reason for this discrepancy is the larger number of species sampled by Walls (2011) and the broader sampling of the Angiosperm phylogeny in her study as compared to our study focusing on 54 species locally co‐occurring in Taiwan. However, both studies sample relatively little of the Angiosperm phylogeny and much denser sampling of the phylogeny would be needed to fully establish whether the lack of signal in our study is representative of the true pattern or not. It is also important to note the decision regarding whether to use phylogenetic comparative methods is contingent on the phylogenetic signal in the focal dataset and not a global sample. Thus, phylogenetic comparative methods were not necessary in the present study despite the findings of Walls (2011). The lack of phylogenetic signal in other traits, such as leaf area, SLA, WD, and Hmax, is consistent with previous work analyzing phylogenetic signal in trait data in five forest plots worldwide (Swenson et al. 2012).

As a result of Kendall's correlation test, three of the leaf vein traits, VLA, DV, and VADR were strongly correlated each other and similarly they were correlated with succulence, wood density, and maximum height (Table 1). The positive wood density – VLA or negative wood density – DV or DAVR relationships suggest that acquisitive leaf and wood trait strategies need not exist in the same individual, and there is potential for species to vary independently along these two important axes of plant functional strategy (Baraloto et al. 2010; Reich 2014). High VLA or low DV species tended to have low leaf succulence values, suggesting a low ability to store water resources as would be expected, on average, given a proportionally smaller areole area. The distance to areole (DA), a measure of average vein thickness, was negatively correlated with specific leaf area (SLA) and mass‐based nitrogen (Nmass), suggesting that species with thicker minor veins tend to have lower SLA and Nmass in the forest studied. VADR, a measure of the size of an areole given the thickness of the veins that supply it, was significantly positively correlated with SLA. Thus, species with relatively small veins per unit areole or larger areole size also tend to have high SLA.

The VLA, DV, and VADR were good predictors of the relative growth rates of species for a wide range of sizes although it is important to note that all correlations between traits and demographic rates were somewhat weak (Fig. 1). This result is in agreement with a recent study reported from 17 dipterocarp species in the common garden in China (Zhang et al. 2015). Specifically, we also found that VLA and DV were not significantly correlated with SLA or Nmass, which are the most commonly measured leaf traits believed to be proxies for photosynthesis. This evidence supports the expectation that alternative and less commonly measured leaf traits also tied to photosynthetic capacity.

Significant correlations between species traits and mortality rates were found only at small sizes (Fig. 2). VLA, VADR, LA, SLA, Nmass, and Pmass were positively correlated, and DA, DV, thickness, and WD were negatively correlated with mortality at small sizes. We did not expect that venation traits would be linked with mortality rates primarily because mortality is likely influenced by multiple factors independent of leaf traits such as mechanical stability, disease resistance, and stochastic events. Our results suggest that leaf venation traits provide an indication of where species fall on the acquisitive–conservative resource‐use axis and these strategies have an influence on the potential mortality rates of small trees. As reported before, for wood density (WD), light wooded species tend to have higher mortality rates (Poorter et al. 2008; Martinez‐Vilalta et al. 2010; Wright et al. 2010; Ruger et al. 2012; Lasky et al. 2013; Iida et al. 2014a,b). The LA, SLA, thickness, Nmass, and Pmass of species were positively related to mortality rates supporting the notion that large, thinner and cheap leaves, and high Nmass and Pmass indicates an acquisitive strategy.

### Leaf venation and soil habitats

We predicted that traits associated with acquisitive resource‐use strategies would be found in species that tend to be associated with edaphic environments that are resource‐rich. In general, we did not find a significant correlation between species traits and average edaphic habitat in this study (Table 2). Wood density was positively correlated with organic carbon. DV was positively correlated with available nitrogen, and VLA and Pmass were negatively correlated with available nitrogen. These results indicate that species traits associated with acquisitive resource‐use strategies such as higher vein density, smaller areoles between veins and lower phosphorus contents of leaves, and low wood density were found in poorer soils. The result that Nmass was positively correlated with available phosphorus was only the case where a trait associated with an acquisitive resource‐use strategy was found in richer soil. Our inconsistent results may partly be influenced by that we used leaves from juvenile trees because trait–edaphic habitat relationship may change during ontogeny. Another possibility is that other resource gradients such as light and water may not be aligned with soil environmental condition related to nutrients such that the distribution of acquisitive strategies is more aligned with these other resource gradients than the edaphic gradient analyzed. The fact that we found only weak trait–edaphic habitat relationships is concordant with a recent study conducted in a subtropical Chinese forest and a tropical Panamanian forest by Liu et al. (2012).

## Conclusion

Establishing linkages between interspecific variations in organismal form and function with demographic performance and species distributions is critical for functional and community ecology. A great deal of research has been conducted over the last decade using easily measured plant leaf functional traits, but particularly in tree ecology, these traits are often poorly associated with demographic rates (e.g., Paine et al. 2015). We believe that these traits are still informative, but they must be considered in the context of the intraspecific variation such as trait–performance associations during ontogenetic development, ultimately entire multivariate phenotype of the organism. In this study, building of recent important syntheses of thought in physiological ecology (e.g., Brodribb et al. 2007; Sack and Scoffoni 2013), we found that leaf venation traits were generally consistently associated with growth rates of demographic performance together with other leaf traits such as succulence and thickness. Each of trait association is weak but the findings provide an additional potential understanding mechanistically linking organismal traits to performance. Further understanding will likely be attained by integrating traits such as leaf venation networks that are mechanistically linked to physiological rates into whole plant allocation strategies and phenotypes and how these relationships change with ontogeny throughout life history.

## Conflict of Interest

None declared.

## Supporting information


**Figure S1.** Correlation coefficients between leaf traits and Relative Growth Rates for large‐statured species.
**Figure S2.** Correlation coefficients between leaf traits and mortality for large‐statured species.

**Table S1.** Species list.

**Table S2.** Phylogenetic signal in twelve species traits.

**Appendix S1.** Detailed description of trait measurements.

**Appendix S2.** Detailed description of demographic models.
