# Supplementary material for: Linking leaf veins to growth and mortality rates: an example from a subtropical tree community
Source: Ecol Evol. 2016 Jul 29;6(17):6085–96. doi: 10.1002/ece3.2311 (PMC5016633; doi:10.1002/ece3.2311)
Supplement: Supplementary file 1 — Figure S1. Correlation coefficients between leaf traits and Relative Growth Rates for large‐statured species. Figure S2. Correlation coefficients between leaf traits and mortality for large‐statured species. [file ECE3-6-6085-s001.doc]

Figure S1. Correlation coefficients between 12 species traits and relative growth rates, *R* at 1 cm stem diameter for large-statured species. Species traits includes, four leaf vein traits of vein density (VLA), the mean distance to nearest areole (DA), the mean distance to nearest vein (DV) and the vein areole distance ratio (VADR), and other 6 leaf traits of leaf area (LA), specific leaf area (SLA), succulence, thickness and mass-based Nitrogen and Phosphorus (Nmass and Pmass), and wood density (WD) and maximum height (Hmax). Each value of correlation coefficient, tau indicates the median of probability distribution of tau. In the case that 95% interval of probability distribution does not include the zero, the correlation between species trait and relative growth rate is significant and then circle was filled. The horizontal line at 20 or 15 shows the number of species compared. We selected largest 20 species based on D95 for comparison (for Nmass and Pmass, 15 species were selected due to lack of trait data).

Figure S2. Correlation coefficients between 12 species traits and mortality rates, *M* at 1 cm stem diameter for large-statured species. Species traits includes, four leaf vein traits of vein density (VLA), the mean distance to nearest areole (DA), the mean distance to nearest vein (DV) and the vein areole distance ratio (VADR), and other 6 leaf traits of leaf area (LA), specific leaf area (SLA), succulence, thickness and mass-based Nitrogen and Phosphorus (Nmass and Pmass), and wood density (WD) and maximum height (Hmax). Each value of correlation coefficient, tau indicates the median of probability distribution of tau. In the case that 95% interval of probability distribution does not include the zero, the correlation between species trait and relative growth rate is significant and then circle was filled. The horizontal line at 20 or 15 shows the number of species compared. We selected largest 20 species based on D95 for comparison (for Nmass and Pmass, 15 species were selected due to lack of trait data).
