# Supplementary material for: Linking leaf veins to growth and mortality rates: an example from a subtropical tree community
Source: Ecol Evol. 2016 Jul 29;6(17):6085–96. doi: 10.1002/ece3.2311 (PMC5016633; doi:10.1002/ece3.2311)
Supplement: Supplementary file 4 — Appendix S1. Detailed description of trait measurements. [file ECE3-6-6085-s004.doc]

**Appendix S1**. Detailed description of trait measurements

*Leaf traits;*

We collected 1 to 3 intact and exposed leaves or leaflets from the outer crown for individual trees (1cm ≤ *D* < 3cm) for 54 species (average 8 trees ranging from 1 to 23 trees per species) and measured leaf area (LA), specific leaf area (SLA), leaf thickness and succulence according to .

The collected leaves were wrapped in wet tissue and were kept in a cool box in the field until they arrived at the laboratory. We measured leaf thickness (mm) using a dial thickness gauge (Dickenmesser, Mitsutoyo, Japan), fresh leaf weight was measured using an electric balance and leaf area was measured using a flatbed scanner in the laboratory within 12 hours after collection. Leaves were then oven-dried for 48 hours at 70°C, and then dry mass was measured. Leaf area (LA; cm^2^) was calculated using the scanned images and image-J (<http://rsb.info.nih.gov/ij/>). Specific leaf area (SLA; cm^2^g^-1^) was calculated as leaf area per dry leaf mass. Leaf succulence (gH_2_O cm^-2^) was calculated as (leaf wet mass – leaf dry mass) / leaf area. We estimated mean values of leaves or leaflets for each species. For 48 of the 54 species, total organic nitrogen mass per unit leaf mass (Nmass, %) and total organic phosphorus mass per unit leaf mass (Pmass, %) were determined by two microplate methods .

*Wood density*, (WD);

Wood density was measured following the Center for Tropical Forest Science (CTFS) wood density measurement protocol ([http://www.ctfs.si.edu/data/documents/Wood_density_draft.pdf](http://www.ctfs.si.edu/data///documents/Wood_density_draft.pdf)). In brief, we randomly selected five individuals of each species outside the 25 ha FDP then used increment borers to extract the wood cores with a 5.1 mm inner diameter. We used increment borers only for trees larger than 6 cm in diameter at breast height. We used water displacement to measure fresh wood volume within 24h after collection, then oven-dried the wood cores to constant weight at 80°C. The wood density was calculated by the dry weight divided by wood volume then weighted by wood segments length. Additional details regarding the methods are discussed in . Wood density across the selected 54 species varied from 0.22 to 0.79 g/cm^3^ with an average of 0.52 g/cm^3^ (Table S1).

*Maximum height (Hmax)*;

Maximum height was estimated as an average value using the heights of the six largest trees of each species within the 25-ha plot. For trees less than 15m height we used a measurement pole and for trees more than 15m in height we used a laser rangefinder (Nikon ProStaff Laser 440) to estimate it height.

*Soil variables within plot*

**We estimated four soil variables, pH in water (pH_water), total soil organic carbon (OC), available nitrogen (AV_N) and available phosphorus (AV_P) in the FDP.** Soil samples were collected from 80 quadrats, which were distributed randomly over the whole 25 ha FDP. Four soil samples were collected for each of the 80 quadrats and mixed for chemical analysis. Available N was extracted by 1 M KCl and measured using the Kjeldhal method . Available P was measured with the Bray No. 1 method . Kriging in the geostatistic software (Surfer 7.0) was used to produce the soil distribution map for the plot on the scale of 20 x 20m.

References

Bremner, J.M. & Keeney, D.R. (1966) Determination and Isotope-Ratio Analysis of Different Forms of Nitrogen in Soils .3. Exchangeable Ammonium Nitrate and Nitrite by Extraction-Distillation Methods. *Soil Science Society of America Proceedings,* **30,** 577-&.

Cornelissen, J.H.C., Cerabolini, B., Castro-Diez, P., Villar-Salvador, P., Montserrat-Marti, G., Puyravaud, J.P., Maestro, M., Werger, M.J.A. & Aerts, R. (2003) Functional traits of woody plants: correspondence of species rankings between field adults and laboratory-grown seedlings? *Journal of Vegetation Science,* **14,** 311-322.

Huang, S.C., Lin, Y.C., Liu, K.F. & Chen, C.T. (2011) Microplate method for plant total nitrogen and phosphorus analysis. *Taiwanese Journal of Agricultural Chemistry and Food Science,* **49,** 19-25.

Iida, Y., Kohyama, T.S., Swenson, N.G., Su, S.H., Chen, C.T., Chiang, J.M. & Sun, I.F. (2014) Linking functional traits and demographic rates in a subtropical tree community: the importance of size dependency. *Journal of Ecology,* **102,** 641-650.

Kuo, S. (1996) Phosphorus. *Methods of Soil Analysis* (ed. D.L. Sparks), pp. 869–919. Agronomy Society of America and Soil Science Society of America, Madison, WI.
