# Supplementary material for: Linking leaf veins to growth and mortality rates: an example from a subtropical tree community
Source: Ecol Evol. 2016 Jul 29;6(17):6085–96. doi: 10.1002/ece3.2311 (PMC5016633; doi:10.1002/ece3.2311)
Supplement: Supplementary file 5 — Appendix S2. Detailed description of demographic models. [file ECE3-6-6085-s005.doc]

**Appendix S2.** Detailed description of demographic models

*Relative growth rate*

We assumed that the RGR of the *i*-th individual tree, *Ri*, is a linear function of the natural logarithm of initial stem diameter *D*1*i* with parameters for species *j* (*rkj,k* =1,2) which is the sum of the community- (*γk,c*) and species-level (*γk,s*). We assumed that *γk,C* has non-informative priors with the Gaussian distribution with a mean of zero and variance of 104, *N*(0, 10-4), whereas *ρk,S* has informative priors in the form of *ρk,S* ~ *N*(0, *τck,r*). For the hyper-prior distribution of *τck,r*, we used a Gamma distribution with both shape and scale parameters set to be 10-2.

*Ri*= *r*1*j* + *r*2*j* ln(*D*1*i*)

The logarithm of the final stem diameter *D*2*i* is assumed to be the sum of the logarithm of initial stem diameter *D*1*i* and, the product of *Ri* and the census interval of tree *i*, *t*­2,*i* – *t*1,*i*.

ln(*D*2*i*) = ln(*D*1*i*) + *R*i (*t*2*i*–*t*1*i*)

We assumed that measurements of stem diameter, *D*1*i* and *D*2*i*, are log-normally distributed with the mean of logarithmic stem diameter with an informative prior. For hyper-prior distribution of an informative prior, we used a Gamma distribution with both shape and scale parameters set to be 10-2.

We calculated growth rates for 61,273 individual trees, which were alive in 2003 and 2008, and estimated the probability distribution of parameters. The probability samples were obtained from three independent Markov chains, in which a total of 400 values were sampled with 150 iteration intervals after a burn-in of 60,000 iterations (in total 120,000 iterations).

------------------------------------------

#Mortality

Our mortality model is based on the observation of a tree individual *i* and whether it survived the census period (*Si* = 1) or not (*Si* = 0). We assumed that *Si* follows a Bernoulli distribution with the predicted probability of survival *pi*

*Si* ~ Bernoulli (*pi*)

Survival probability *pi* of an individual tree *i*, is calculated from its predicted annual per capita mortality rate constant (*Mi*, year-1) of individual tree *i* and adjusted for the time period between the first census (*t*1) and the second census (*t*2)

*pi* = exp[−*Mi*(*t*2*i*-*t*1*i*)],

The mortality rate, *Mi* is predicted from a function of the stem diameter *Di* of individual *i* at the first census, *D*1*i*, and a species-specific parameter *mkj* (*k* = 1, 2, 3) of species *j*.

ln(*Mi*) = *m*1*j* + *m*2*j* * ln(*D*1*i*) +*m*3*j* * *D*1*i*

We assumed that all parameters are the sum of the community-level (*μk,c*) and species-level (*μk,s*). We assumed that μ*k,c* had non-informative priors that had the form *N*(0, 10-4), while *μk,s* had informative priors in the form, *μk,s* ~ *N*(0, *μk*). For the hyper-prior distribution of *μk*, we used a Gamma distribution with both shape and scale parameters set to be 10-2.

We used census data of 75,071 individual trees that were alive in 2003 and recensused in 2008, and estimated the probability distribution of the parameters. The probability samples were obtained from three independent Markov chains, in which a total of 400 values were sampled with 75 iteration intervals after a burn-in of 45,000 iterations (in total 75,000 iterations). Sampling from the posterior distribution of all parameters was performed using the Markov chain Monte Carlo method with WinBUGS 1.4.3 . The convergence of the Markov chains was checked with $\hat{R}$ for each parameter by comparing the variance within each chain and among chains. $\hat{R}$ is the potential scale reduction factor at convergence (R hat ≈ 1).

# *References*

Gelman, A., Carlin, J.B., Stern, H.S. & Rubin, D.B. (2003) *Bayesian Data Analysis,* 2nd edn. Champan & Hall, London.

Spiegelhalter, D.J., Thomas, A., Best, N.G. & Lunn, D. (2003) WinBUGS version 1.4 user manual.MRC Biostatics Unit, Cambridge.
